# Interleukins, growth factors, and transcription factors are key targets for gene therapy in osteoarthritis: A scoping review

**DOI:** 10.3389/fmed.2023.1148623

**Published:** 2023-04-03

**Authors:** Melanie Uebelhoer, Cécile Lambert, Juliane Grisart, Kilian Guse, Stanislav Plutizki, Yves Henrotin

**Affiliations:** ^1^Artialis S.A., Liège, Belgium; ^2^musculoSKeletal Innovative Research Lab (mSKIL), Center for Interdisciplinary Research on Medicines, University of Liège, Liège, Belgium; ^3^GeneQuine Biotherapeutics GmbH, Hamburg, Germany; ^4^Department of Physical Therapy and Rehabilitation, Princess Paola Hospital, Vivalia, Marche-en-Famenne, Belgium

**Keywords:** genetic therapy, osteoarthritis, gene transfer techniques, interleukins, growth factors, transcription factors

## Abstract

**Objective:**

Osteoarthritis (OA) is the most common degenerative joint disease, characterized by a progressive loss of cartilage associated with synovitis and subchondral bone remodeling. There is however no treatment to cure or delay the progression of OA. The objective of this manuscript was to provide a scoping review of the preclinical and clinical studies reporting the effect of gene therapies for OA.

**Method:**

This review followed the JBI methodology and was reported in accordance with the PRISMA-ScR checklist. All research studies that explore *in vitro, in vivo*, or *ex vivo* gene therapies that follow a viral or non-viral gene therapy approach were considered. Only studies published in English were included in this review. There were no limitations to their date of publication, country of origin, or setting. Relevant publications were searched in Medline ALL (Ovid), Embase (Elsevier), and Scopus (Elsevier) in March 2023. Study selection and data charting were performed by two independent reviewers.

**Results:**

We found a total of 29 different targets for OA gene therapy, including studies examining interleukins, growth factors and receptors, transcription factors and other key targets. Most articles were on preclinical *in vitro* studies (32 articles) or *in vivo* animal models (39 articles), while four articles were on clinical trials related to the development of TissueGene-C (TG-C).

**Conclusion:**

In the absence of any DMOAD, gene therapy could be a highly promising treatment for OA, even though further development is required to bring more targets to the clinical stage.

## 1. Introduction

Osteoarthritis (OA) is the most common degenerative joint disease (1). Traditionally considered as a disease of “wear and tear”, OA is now considered as a complex disorder affecting the whole joint and involving pro-inflammatory immune pathways (2, 3). For the patients, it is associated with a significant handicap and alteration of their quality of life. Currently, this pathology affects 500 million people worldwide and therefore contributes highly to the costs of the health and social care (4, 5).

Despite the high prevalence of OA, there is no treatment to cure or delay OA. Currently, medical treatment focuses on symptoms relief with painkillers and anti-inflammatory drugs (6, 7). Recommendations for OA non-surgical treatments are divided into non-pharmacological and pharmacological interventions with the purpose to reduce pain and joint stiffness and maintaining and improving mobility. The pharmacological treatments are dependent on patients' preferences, its phenotype, the severity of the diseases, and the presence of co-morbidities (8). In the absence of a disease-modifying OA drug (DMOAD), clinical guidelines suggest physical therapy, education, and weight management as a core treatment with pharmacological intervention if needed (9, 10).

The development of an effective treatment for OA is highly challenging. Recent advances have been made in the development of a range of biological drugs that position gene therapy as a promising option to overcome the limitations of traditional therapeutics in OA (11, 12). Gene therapy has the advantage of local delivery and, therefore, local production of therapeutic proteins for targeted, local treatment of the joint, along with a potentially reduced risk of systemic adverse events and drug-drug interactions (13). In addition, relatively long-term expression of the target gene can be achieved, thus avoiding repeated intra-articular (IA) injections.

While viral gene transfer is more efficient, non-viral gene delivery is considered safer (14, 15). It can involve lipid-based systems, polymers, nanoparticles, natural components, or simple plasmids (14, 15). Viral therapy, on the other hand, involves the administration of viral vectors, such as adenovirus (Ad), helper-dependent adenovirus (HDAd or HDV), adeno-associated virus (AAV), and retroviruses (RV) including lentivirus (LV) (13). Both non-viral and viral gene delivery systems can be used for direct *in vivo* administration or for cell-based approaches in which cells are genetically modified *ex vivo* and then infused into the patient. In cell-based approaches, different cell types can be used such as synovial fibroblasts, primary chondrocytes, mesenchymal stem cells (MSCs) or pluripotent stem cells. The investigated transgenes include both secreted proteins such as growth factors and anti-inflammatory proteins, as well as transcription factors, components of signaling pathways and small regulatory nucleic acids (miRNAs).

Gene therapy for OA treatment is a booming research topic, as evidenced by an explosion in the volume and in the quality of genomic studies, a growing number of preclinical and clinical-stage gene therapy drug candidates, and the first gene therapies being tested in clinical trials (11, 12). While a significant number of reviews of the literature exist about gene therapy in OA, no scoping reviews have thus far been published on this subject. We, therefore, propose this scoping review to map the relevant literature related to both non-viral and viral gene therapy in the management of OA.

The objective of this scoping review was to provide a comprehensive view on the current knowledge about gene therapies developed in the context of preclinical and clinical studies targeting OA. Four sub-questions were developed and will reflect the objectives of the scoping review:

1. What are the delivery methods for gene therapies in OA?
2. Which models have been developed to assess gene therapies in OA?
3. What are the target genes in gene therapy approaches for OA?
4. What are the effects observed for gene therapies in OA?

## 2. Methods

### 2.1. Eligibility criteria

**Population:** All research studies that address OA disease were included.

**Concept:** All research studies that explore *in vitro, in vivo*, or *ex vivo* gene therapies as well as clinical trials on gene therapies that follow a viral or non-viral gene therapy approach were considered. Gene therapies using siRNA approaches, or targeting miRNA, circRNA, as well as germline gene therapies including CRISPR/Cas9, were not taken into consideration.

**Context:** All research studies that include models of experimental OA, *in vitro* and *in vivo* models of OA, as well as clinical trials and studies on humans were included.

**Types of sources:** This scoping review considered all peer-reviewed published research studies that address the use of gene therapy in OA. Only studies published in English were considered for inclusion in this review. There were no limitations to their date of publication, country of origin, or setting.

The protocol for this review has not been registered.

### 2.2. Information sources

The following electronic databases were searched: Medline ALL Ovid, Embase, and Scopus. The most recent search was executed on March 7, 2023.

### 2.3. Search

The research team (MU and CL) undertook a preliminary search to identify controlled terms and keywords in titles and abstracts from relevant literature. Then, an extensive literature search was conducted.

The search strategies (see below for an example of Ovid MEDLINE and Appendix) were performed with the help of an information specialist experienced in evidence synthesis and adapted for each database. The search strategies focused on two concepts—gene therapy and osteoarthritis—and used a set of keywords and controlled terms.

Database: Ovid MEDLINE (R) ALL <1946 to March 02, 2023>

Search Strategy:

1. Genetic Therapy/ (52544)
2. Targeted gene repair/ (201)
3. ((gene or genes or genetic) adj3 (therap^*^ or repair^*^ or correction^*^)).ti,ab,kf. (82728)
4. DNA therap^*^.ti,ab,kf. (100)
5. 1 or 2 or 3 or 4 (104404)
6. exp Osteoarthritis/ (75797)
7. osteoarthr^*^.ti,ab,kf. (92344)
8. osteo-arthr^*^.ti,ab,kf. (635)
9. arthros^*^.ti,ab,kf. (46096)
10. (degenerative adj3 arthriti^*^).ti,ab,kf. (1748)
11. (degenerative adj3 joint^*^ adj3 disease^*^).ti,ab,kf. (3757)
12. coxarthros^*^.ti,ab,kf. (1705)
13. gonarthros^*^.ti,ab,kf. (1221)
14. 6 or 7 or 8 or 9 or 10 or 11 or 12 or 13 (155956).
15. 5 and 14 (450).

### 2.4. Selection of sources of evidence

All identified relevant records were uploaded into Covidence (https://www.covidence.org) and duplicates were removed. Following a pilot test, titles and abstracts were screened by two independent reviewers (MU & CL) for assessment according to the inclusion criteria for the scoping review. Then, the full text of selected papers was assessed according to the inclusion criteria by the same two independent reviewers. For both stages, discrepancies between the reviewers were resolved by discussion or by consulting a third reviewer (YH).

### 2.5. Data charting process

A data-charting form was jointly developed by two reviewers (MU & CL) to determine which variables to extract (experimental models, delivery methods, targets, and effects). This data charting form was used to extract data from selected studies. The same two reviewers independently charted the data, discussed the results, and updated, if necessary, the data-charting form to enable the capture of all relevant data to answer the review question.

### 2.6. Data items

Data on the population, concept, context, and key findings relevant to the review question was extracted. The data-charting form included the following items:

- Author and year of publication
- Objectives
- Participants (for clinical trials, only)
- Study design
- Disease models
- Delivery methods
- Targets
- Effects.

### 2.7. Synthesis of results

Data were analyzed and summarized quantitatively through numerical counts as well as descriptively. Data were presented graphically or in tabular form. A narrative summary accompanied the charted and/or tabulated results and describes how the results relate to the objectives and questions of the review.

## 3. Results

### 3.1. Selection of sources of evidence

**Figure 1:**
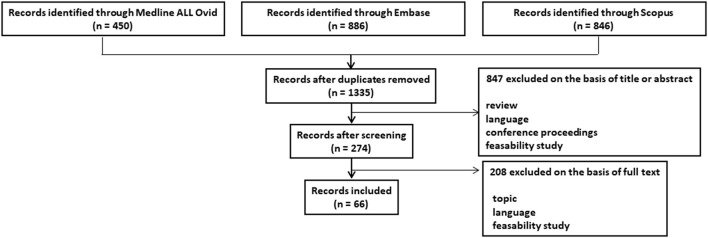
PRISMA flowchart outlining study selection procedure.

The PRISMA flowchart outlining study selection is shown in Figure 1. An initial search identified 2,182 studies, 1,335 of which remained after the removal of duplicates. All but 274 of these studies were excluded at the stage of abstract review. Furthermore, 208 additional studies were excluded after reviewing the full manuscript. The reasons for exclusion at each stage are detailed in Figure 1. Overall, 66 studies responded to the inclusion criteria and constituted the study data.

### 3.2. Characteristics of sources of evidence

Most articles were on preclinical *in vitro* studies (32 articles) or *in vivo* animal models (39 articles), while four articles were on clinical trials.

### 3.3. Results of individual sources of evidence

**Figure 2:**
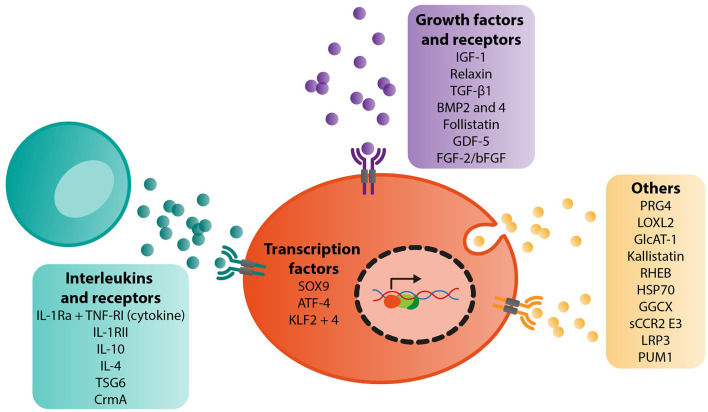
Summary of target genes identified for gene therapy of OA and their biological functions.

We found a total of 29 different targets for gene therapy in the context of OA. These included studies examining interleukins (IL-1Ra alone or in combination with another target, TNF-RI, IL-1RII, IL-10, IL-4, TSG6, CrmA), growth factors, and receptors (IGF-1, relaxin, TGF-β1, BMP2 and 4, follistatin, GDF-5, FGF-2/bFGF), transcription factors (SOX9 alone or in combination with another target, KLF2 and 4, and ATF-4) and other key targets such as PRG4 (alone or in combination with another target), LOXL2, GlcAT-1, GGCX, kallistatin, RHEB, HSP70, PUM1, sCCR2 E3 and LRP3 (Figure 2).

## 4. Discussion

### 4.1. Summary of evidence

#### 4.1.1. Interleukins

The activation of the immune system is closely linked to the initiation and perpetuation of low-grade systemic inflammation in OA (3). Interleukin-1 (IL-1) is considered among the most powerful molecules of the innate immune system and is linked to the pathogenesis of OA. It therefore appears to be a natural target for gene therapy. The IL-1 family is constituted of seven agonists including IL-1α and β, and four antagonists including the IL-1 receptor antagonist (IL-1Ra). The effect of IL-1 on joint tissues can be controlled by inhibiting its receptors using gene therapy-mediated overexpression of IL-1Ra (Table 1).

**Table 1:** Interleukins.

**References**	**Objectives**	**Study design**	**Disease models**	**Delivery methods**	**Targets**	**Effects**
Pelletier et al. (16)	Evaluate the therapeutic effect of IL-1Ra on progression of OA lesions	Preclinical *in vivo*	Dog ACLT section and partial synovectomy of the knee	Retrovirally transduced synovial fibroblasts	IL-1Ra	Reduced progression of experimentally induced OA lesions after intraarticular injection of transduced synovial cells
Baragi et al. (17)	Demonstrate the chondroprotective effect of IL-1ra	Preclinical *in vitro* and *ex vivo*	Human articular OA chondrocytes and OA cartilage explants	Adenovirus: Ad.RSV hIL-1ra	IL-1Ra	Adherence and viability of transduced chondrocytes on surface of hyaline cartilage; protection of OA cartilage from Il-1β-induced matrix degradation
Nixon et al. (18)	Investigate the disease-modifying properties of IL-1Ra gene therapy	Preclinical *in vivo*	Mouse ACLT model	Helper-dependent adenovirus: HdAd-mIL-1Ra	IL-1Ra	Prevention and treatment of surgically induced OA: improved histologic scores, fewer osteophytes; higher cartilage volume and surface
			Horse osteochondral fragment model	Helper-dependent adenovirus: HdAd-eqIL-1Ra		Improvement in pain: improved lameness, range of motion and effusion; better cartilage status; improved synovial membrane status; fewer osteophytes
Deng et al. (19)	Evaluate the potential of loaded nanomicelles to treat articlular inflammation in *in vivo* TMJOA model	Preclinical *in vivo*	Rat MIA model	Polyplex nanomicelles	IL-1Ra	Reduced pain behavior; less cartilage degradation; lower Mankin score; reduced surface fibrosis; reduced OA progression; downregulation of pro-inflammatory cytokines
Senter et al. (20)	Assess efficacy, biodistribution and safety of HDAd-ratIL-1Ra as well as biodistribution of FX201 (human equivalent)	Preclinical *in vivo*	Rat ACLT model	Helper-dependent adenovirus: HDAd-ratIL-1Ra and FX201 (HDAd-hIL-1Ra)	IL-1Ra	HDAd-ratIL-1Ra decreased OA-induced joint damage; HDAd-ratIL-1Ra and FX201 mainly localized to knee joint; HDAd-ratIL-1Ra well-tolerated
Fernandes et al. (21)	Determine the effect of IL-1Ra through a lipoplex on structural changes in *in vivo* OA model	Preclinical *in vivo*	Rabbit menisectomy model	IL-1Ra plasmid (Lipoplex)	IL-1Ra	Reduced width of osteophytes and size of macroscopic lesions (dose-dependent); reduced severity of histologic cartilage lesions; presence of IL-1Ra in the synovium and cartilage of injected rabbits
Zhang et al. (22)	Evaluate the efficiency of chitosan-EGFP nanoparticles for gene therapy of OA	Preclinical *in vivo*	Rabbit medial collateral ligament excision and medial menisectomy model of OA	Chitosan-EGFP nanoparticles	IL-1Ra or IL-10	Less severe lesions after treatment with IL-1Ra; (no expression of IL-10, therefore effect was not studied)
Deng et al. (23)	Development of a new nanoparticle made of chitosan (CS)/hyaluronic acid (HA)/plasmid-DNA	Preclinical *in vitro*	Rat IL-1β-treated synoviocytes	CS/HA/pDNA nanoparticles	IL-1Ra	Increased IL-1Ra expression and decreased MMP-3, MMP-13, COX-2 and iNOS expression in IL-1β-induced synoviocytes
Frisbie et al. (24)	Evaluate the utility of the equine IL-1Ra gene therapy in a equine OA model	Preclinical *in vitro*	Equine synoviocytes	Adenovirus: Ad-EqIL-1a	IL-1Ra	Dose dependent increase of IL-1Ra after transduction of equine synoviocytes; inhibit PGE2 production in response to human IL-1α
		Preclinical *in vivo*	Equine OA model			Improvement in clinical parameters of pain, disease activity, preservation of articular cartilage, beneficial effects on histologic parameters of synovial membrane and articular cartilage
Goodrich et al. (25)	scAAVIL-1Ra dosing trial in an equine model	Preclinical *in vivo*	Skeletally mature horses	Adenoassociated virus: scAAV2IL-1ra	IL-1Ra	Transduction of the scAAV vector both in the synovial and cartilage tissues; no evidence of intra-articular toxicity; neutralizing ABs within 2 weeks of administration which persisted for the duration of the study but did not lower protein expression intra-articularly
Watson Levings et al. (26)	Generate pharmacokinetic profile of homologous gene delivery of scAAV.IL-1Ra	Preclinical *in vivo*	Naturally occurring OA in horses	Adenoassociated virus: sc-AAV.eqIL-1Ra	IL-1Ra	Safe and sustained drug delivery to joints
Watson Levings et al. (27)	Efficacy of local treatment with scAAV.IL-1Ra	Preclinical *in vivo*	Horse surgically induced osteochondral fragmentation (OCF) model	Adenoassociated virus: sc-AAV.eqIL-1Ra	IL-1Ra	Reduced forelimb lameness; reduced inflammation; enhanced repair of osteochondral injury; reduced joint effusion; reduced synovial proliferation
Glass et al. (28)	Evaluate the possibility to combine gene therapy and functional tissue engineering to develop engineered cartilage with inductible immunomodulatory properties	Preclinical *in vitro*	IL-1β-stimulated MSCs from human bone marrow	IL-1Ra lentivirus *via* scaffold	IL-1Ra	Engineered cartilage constructs are capable of inducible and tunable IL-1Ra production at therapeutically relevant concentrations; these constructs protect from the effects of IL-1
Gabner et al. (29)	Evaluate IL-1Ra expression in equine MSCs	Preclinical *in vitro*	Equine OA chondrocytes co-culture	Lentivirus: pSEWNFKBIL-1Ra	IL-1Ra	Protective ability of the IL-1Ra protein (increased ACAN and COL2A1 and decreased IL-6, MMP-1 and MMP-13); upon TNF-α, a dose-dependent increase in IL-1Ra expression in MSC/IL-1Ra cells
Chen et al. (30)	Investigate the combinatorial effect of adenovirus-mediated overexpression of bFGF vs. IL-1Ra vs. IGF-1 on OA	Preclinical *in vitro*	Human articular OA chondrocytes	Adenovirus: AdbFGF; AdIL-1Ra; AdIGF-1	bFGF •IL-1Ra •IGF-1	Increased chondrocyte proliferation; increased GAG and type II collagen synthesis
		Preclinical *in vivo*	Rabbit ACLT model			Protects from cartilage degradation: lower Mankin score; increased type II collagen and proteoglycan synthesis; better results with combinations
Zhang et al. (31)	Evaluate the effect of using IL-1Ra and IL-10 together as gene therapy for OA	Preclinical *in vivo*	Rabbit medial collateral ligament excision and medial menisectomy model of OA	Retrovirus: PLXRN-IL-1Ra and PLXRN-IL-10	IL-1Ra and IL-10	Reduced cartilage lesions and decreased loss of proteoglycans after combined injection; no effect on synovitis
Haupt et al. (32)	Evaluate the combinatorial effect of adenovirus-mediated overexpression of IGF-1 and IL-1Ra in an OA culture model	Preclinical *in vitro*	IL-1β-stimulated horse cartilage explants and synovial membrane	Adenovirus: equine AdIGF-1; equine AdIL-1Ra	IGF-1 and IL-1Ra	Matrix synthesis stimulation and catabolics blockers, prevention matrix degradation by IL-1, protection and partial restoration of cartilage matrix
Zhang et al. (33)	Evaluate feasibility of gene therapy by co-injecting IL-1Ra and TGF-beta1 genes into joints together with liposomes	Preclinical *in vivo*	Rabbit medial collateral ligament excision and medial menisectomy model of OA	Lipofectamine transfection	IL-1Ra and TGF-β1	Inhibited cartilage damage and prevention of osteophyte formation; increased Mankin score; normalization of choncdrocyte number and order; increased type II collagen expression and ECM deposition
Wang et al. (34)	Determine the efficacy of local expression of IL-1Ra and sTNF-RI	Preclinical *in vivo*	Rabbit medial collateral ligament excision plus medial menisectomy OA model	Adenovirus: Ad-IL-1Ra and Ad-sTNF-RI	IL-1Ra and TNF-RI	Reduced cartilage lesions after IL-1Ra injection and combination, but not after sTNF-RI injection alone; reduced synovitis after combinatorial injection
Attur et al. (35)	Determine the effect of IL-1RII expression on modulating effects of IL-1beta	Preclinical *in vitro*	Human articular OA chondrocytes and synoviocytes	Adenovirus: AdRSVRII	IL-1RII	Dose-dependent decrease in response of OA chondrocytes and synoviocytes to IL-1beta (induction of NO, PGE2, IL-6, IL-8; production of IL-1beta and proteoglycan) protection of other cells in co-culture and transplant from effect of IL-1beta *via* sIL1-RII
Broeren et al. (36)	Determine the therapeutic potential of CXCL10p-IL10 in 3D micromass synovial membrane model that mimics early stage OA	Preclinical *in vitro*	Human OA synovial tissue	Lentivirus: CXCL10p-IL10	IL-10	Reduced IL-1β-induced secretion of IL-1β and IL-6
Farrell et al. (37)	Evaluate the ability of hMSCS overexpressing vIL-10 to modulate the inflammation and alter OA disease progression	Preclinical *in vivo*	CIOA mouse model	Adenovirus: AdIL-10	vIL-10	A trend toward more damage in animals treated with hMSCs; reduced CD4 and CD8 T cells in the vIL-10-expressing hMSC group
Cameron et al. (38)	Investigate combinatorial effect of BM-MSCs and IL-10 overexpression	Preclinical *in vitro*	IL-1β/TNF-α-stimulated horse BM-MSCs and cartilage explant co-cultures	Adenoassociated virus: AAV-IL10	IL-10	Decreased T cell proliferation; decreased expression of inflammatory markers (IL-1beta, IL-6 and TNF-alpha) in stimulated cartilage explant co-cultures; no protection from ECM degradation
Watkins et al. (39)	Toxicology study of intra-articular hIL-10var pDNA in dogs	Preclinical *in vivo*	Healthy naive dogs	hIL-10var pDNA (transfection with Fugene 6)	IL-10	Well-tolerated without toxicologic effects for up to 1.5 mg of plasmid
	Efficacy of intra-articular hIL-10var pDNA in companion dogs		Naturally occurring OA in companion dogs			No adverse changes; decreased pain scores
Lang et al. (40)	Optimization of a non-viral transfection system to evaluate Cox-2 controlled IL-4 expression for OA gene therapy	Preclinical *in vitro*	Equine chondrocytes	pN3.Cox2.IL-4 (different transfection agents)	IL-4	Exogenous stimulation of chondrocytes transfected with pN3.Cox-2.IL-4 led to increased IL-4 expression and decreased IL-1β,−6,−8, MMP-1 and−3 expression
Song et al. (41)	Investigate whether IL-4 transfection and spheroid formation potentiates therapeutic effect of MSCs for OA	Preclinical *in vitro*	Rat IL-1β stimulated primary chondrocytes	IL-4 MSC spheroids (delivered *via* cationic liposomes)	IL-4	Reduced IL-1beta induced apoptosis; lower production of osteoarthritic factors; higher production of cartilage ECM
		Preclinical *in vivo*	Rat ACLT-MMx model			Enhanced attenuation of tissue regeneration; improved chondroprotective and anti-inflammatory effects; higher pain relief
Broeren et al. (42)	Determine the effect of viral overexpression of TSG-6 in experimental OA	Preclinical *in vitro*	BM-derived cells differentiated into osteoclasts	Adenovirus: pShuttle-CMV-TSG-6	TSG-6	Inhibited osteoclast activity
		Preclinical *in vivo*	Mouse CIOA model			No difference in protease activity or cartilage damage; increased ectopic bone formation
Qiu et al. (43)	Investigate the effect of HA/CS/pCrmA on OA synoviocytes	Preclinical *in vitro*	IL-1β stimulated primary rat synoviocytes	Hyaluronic acid/chitosan (HA/CS) nanoparticles	pCrmA	Attenuated IL-1β mediated inflammation: normalization of increased MMP-3 and MMP-13 expression caused by IL-1β stimulation

##### 4.1.1.1. IL-1Ra

The therapeutic effect of IL-1Ra on the progression of OA lesions was evaluated by Pelletier et al. in a surgical dog OA model (ACLT model) (16). Interestingly, they observed a reduction in macroscopic lesion severity in animals that were injected with synovial cells retrovirally transduced with the IL-1Ra gene compared with the lac Z (control) group. Similarly, Baragi et al. observed a positive effect on human OA articular chondrocytes and OA cartilage explants transduced with Ad.RSV hIL-1ra cDNA (17). Nixon et al. investigated the disease-modifying properties of IL-1Ra gene therapy with an adenoviral-mediated overexpression of IL-1Ra under the control of the inflammation-inducible NF-kB promoter (HDAd-IL-1Ra) (18). In a anterior cruciate ligament transection (ACLT) mouse model of OA, they highlighted an improvement in histological scores, a decrease in osteophytes, and an increase in the volume and surface area of the cartilage. HDAd-IL-1Ra treatment also protected osteoarthritic mice against increased thermal hyperalgesia. Moreover, they confirmed these results in an osteochondral fragment horse OA model, observing an improvement in pain, claudication, and amplitude of movement, together with improved macroscopic and histological cartilage and synovium status. Deng et al. evaluated the potential of loaded nanomicelles to treat articular inflammation in a rat temporomandibular joint OA model (MIA model) (19). In this one, IL-1Ra mRNA reduced pain behavior and attenuated cartilage degradation as evidenced by the decrease of Mankin score. In addition, authors observed a downregulation of pro-inflammatory cytokines (IL-6 and TNF-α) blocking the MIA-induced inflammatory cascade. Finally, Senter et al. evaluated efficacy, biodistribution and safety of FX201 (HDAd expressing human IL-1Ra) and its ratIL-1Ra expressing ortholog vector in the rat ACLT OA model (20). IL-1Ra gene therapy significantly reduced joint damage in arthritic animals, and the gene therapy vectors were shown to be well-tolerated and to remain predominantly at the injection site after IA injection. Following these investigational new drug (IND)-enabling studies, a Phase 1 clinical trial with FX201 was conducted in 72 knee OA patients (NCT04119687).

Fernandes et al. developed a direct method using a non-viral vector, named “lipoplex” (21). It is a plasmid complexed to a lipid injected IA into the knees of rabbits. In a rabbit menisectomy model, they observed a dose-dependent reduction of lesion size associated with a reduction of the width of osteophytes. Another group, Zhang et al., evaluated a different non-viral strategy, in a rabbit surgery OA model (medial collateral ligament excision and medial menisectomy) (22). They demonstrated that the transfection efficiency of chitosan–DNA nanoparticles containing IL-1Ra or IL-10, was closely related to the gene product, and observed less severe cartilaginous lesions after treatment with IL-1Ra. Deng et al., employed a non-viral strategy using nanoparticles consisting of chitosan (CS)/hyaluronic acid (HA)/plasmidDNA (pDNA) encoding IL-1Ra to transfect primary synoviocytes (23). This strategy reduced MMP-3,−13, COX-2, and iNOS expression in IL-1β-stimulated synoviocytes. These results constitute a promising therapeutic approach for synovitis. Frisbie et al. investigated the effect of an Ad vector expressing IL-1Ra (Ad-EqIL-1Ra) in an equine osteochondral fragment OA model (24). They demonstrated that in addition to the conservation of the articular cartilage and the synovial membrane, the IA injection of the Ad-EqIL-1Ra vector significantly improved pain- and inflammation-related parameters. Goodrich et al. worked with an equine model, with the aim of developing the self-complementary AAV (scAAV) to produce higher levels of protein more quickly than with single stranded AAV (25). They proposed a dosing/alternative serotype redosing protocol, and examined the neutralizing antibody (Nab) response to the capsid. They did not observe any IA toxicity but the development of Nab against AAV capsid, which did however not decrease protein expression. Watson Levings et al. investigated the therapeutic capacity of an scAAV (sc-AAV.eqIL-1Ra) in two equine OA models (26, 27). On the first spontaneous model, they confirmed the safety. On the second, a surgically induced osteochondral fragmentation model of early OA, the results showed both a reduction in forelimb lameness and a reduction in inflammation. The authors also observed an improvement in the repair of osteochondral lesions, a reduction in joint effusion, and synovial proliferation. These data confirmed the results of previous studies.

Besides the “traditional” methods, Glass et al. investigated the possibility to combine gene therapy and tissue engineering by inducing IL-1Ra overexpression in human MSCs *via* scaffold-mediated lentiviral gene delivery (28). The results were quite promising since they demonstrated that the constructs could produce IL-1Ra in an inducible manner and that they protected against the effects of IL-1α. Gabner et al. also investigated the combination of tissue engineering methods employing bone marrow-derived equine MSCs transduced with a lentiviral vector expressing IL-1Ra gene under the control of the inducible NF-kB promoter (pSEWNFKBIL-1Ra) (29). They observed an increase of aggrecan, collagen IIA1 expression and a decrease of IL-6,−8, MMP-1, and−13 expression in equine chondrocytes stimulated with IL-1β or TNF-α.

Based on the assumption that the therapeutic effects of a single gene administration are limited, several teams have also evaluated the combinatorial effect of different targets. For example, Chen et al. investigated the combination of basic fibroblast growth factor (bFGF)/IL-1Ra and/or insulin-like growth factor 1 (IGF-1) (ADbFGF/ADIL-1Ra and/or ADIGF-1) (30). Transfection of human OA chondrocytes with two or three genes in different combinations resulted in increased proliferation of chondrocytes along with an increased synthesis of glycosaminoglycans, type II collagen, and TIMP-1, and a decreased expression of MMP-3,−13 and ADAMTS-5. In a rabbit ACLT model, the same team observed similar effects for type II collagen as well as a lower cartilage Mankin score. For his part, Zhang et al. evaluated the effect of IL-1Ra/IL-10 using retroviruses PLXRN-IL-1Ra and PLXRN-IL-10 in a rabbit OA model (medial collateral ligament excision and medial menisectomy) (31). While they observed no effect on synovitis, results on cartilage damage and glycosaminoglycan (GAG) content were promising. Haupt et al. tested the combinatorial effect of adenovirus-mediated overexpression of IL-1Ra with IGF-1 (equine AdIL-1Ra and equine AdIGF-1) to control cartilage degradation (32). *In vitro*, they demonstrated that the gene transfer promoted GAG and type II collagen synthesis and reduced IL-1β, IL-1α, and matrix metalloproteinases expression. Zang et al. evaluated the combined injection of IL-1Ra and TGFβ1 with liposomes *in vivo* (33). In a rabbit OA model (medial collateral excision and medial menisectomy), they observed a significant inhibition of cartilage matrix degradation as well as a prevention of osteophyte formation. The Mankin score was decreased in the transfected groups, and the expression of IL-1Ra and TGF-β1 was correlated to an increase of type II collagen expression and an extracellular matrix deposition. Finally, Wang et al. considered IL-1Ra with the soluble tumor necrosis factor-α receptor type I (sTNF-RI) (34). In a rabbit surgery OA model (Medial collateral ligament excision and medial menisectomy), IA injection of the combination Ad-IL-1Ra and Ad-sTNF-RI resulted in a decrease in cartilage lesions and synovitis whereas injection of sTNF-RI alone had no significant effect on these parameters. Multiple target combinations therefore seem to have beneficial effects in the context of gene therapy in OA.

Taken together, IL-1Ra gene therapy has been reported to improve clinical parameters such as pain and disease activity. In addition, beneficial effects on histological parameters of the synovial membrane and articular cartilage have been observed by different study groups.

##### 4.1.1.2. IL-1RII

Type II IL-1β receptor (IL-1RII) serves as a “decoy” target for IL-1β. Previous data showed that it significantly inhibited IL-1-induced production of NO and/or PGE_2_ in synovial cells, chondrocytes, and epithelial cells. Under OA conditions, these same cells lack detectable amounts of IL-1Ra and IL-1RII, two molecules that normally antagonize IL-1 (44). In this context, and in order to reconstitute the functional expression of IL-1RII, Attur et al. transduced human articular OA chondrocytes and synoviocytes (IL-1RII^−^ cells) with an Ad vector expressing IL-1RII, AdRSVRII (35). They observed a dose-dependent decrease of NO, PGE_2_, IL-6, IL-8, IL-1, and proteoglycan production in response to IL-1β. Another consequence was the release of sIL-1RII from transduced cells, thus protecting the other cells in co-culture and in transplants from the effect of IL-1β *via* sIL1-RII.

##### 4.1.1.3. IL-10

Another interesting target for gene therapy is the cytokine IL-10. Produced by cells of innate and adaptive immunity, it is known for its anti-inflammatory and immunosuppressive properties. Thus, several gene therapies targeting this pleiotropic cytokine have been reported. In a rabbit OA model (medial collateral ligament excision and medial menisectomy), Zhang et al. were the first to evaluate the effect of retroviral IL-1Ra and IL-10 gene delivery on rabbit knee joints during the early inflammatory phase of OA (31). Thus, IA retroviral administration of IL-1Ra and IL-10 (PLXRN-IL-1Ra and PLXRN-IL-10) was able to impede an acute inflammatory reaction. IL-1Ra was more effective than IL-10 and most importantly, the co-injection of IL-1Ra and IL-10 was found to have significantly greater chondroprotective effects. Cartilage degradation and loss of proteoglycans was significantly more reduced by the combination than either one alone. In a 3D micromass synovial membrane model that mimics early-stage OA, Broeren et al. showed that adequate amounts of IL-10 transgene (lentivirus named CXCL10p-IL-10) reduced the synovial production of IL-1β and IL-6 and consequently, the inflammatory response (36). Farrell et al. assessed whether hMSCs overexpressing vIL-10 (*via* AdIL-10) were able to modify inflammation and adjust OA progression in a collagenase induced osteoarthritis model (CIOA) mouse model (37). Interestingly, the amount of activated CD4 and CD8 T-cells was significantly reduced in the vIL-10-expressing hMSCs group. A subsequent study conducted by Cameron et al. supports their findings (38). Indeed, in a stimulated, co-culture OA model, T-cell proliferation was significantly reduced by BM-MSCs overexpressing IL-10 (AAV-IL-10). This was accompanied by a reduced expression of inflammatory markers (IL-1β, IL-6, and TNF-α). Finally, considering the short half-life of IL-10 as well as its poor joint permeability, Watkins et al. used a plasmid DNA-based therapy for the production of a long-acting human IL-10 variant called hIL-10var (39). The first results of the 6-month GLP toxicology study looked promising. Bilateral IA injections of up to 1.5 mg of hIL-10var pDNA into canine stifle joints were well-tolerated and without pathologic findings. In addition, they have also conducted a small double-blinded, placebo-controlled study to assess the effect of IA hIL-10var pDNA on pain in companion dogs with naturally occurring OA, and observed a decrease of pain parameters without any adverse findings.

In summary, IL-10 gene therapy mainly reduces the expression of inflammatory markers. Protection from ECM degradation has only been reported in combination with other target genes.

##### 4.1.1.4. IL-4

Among the anti-inflammatory cytokines, IL-4 is considered to have a strong therapeutic potential due to its inhibitory effect on IL-1β, the main mediator of inflammation leading to cartilage degradation. In this context, Lang et al. developed a non-viral transfection model to assess Cox-2 regulated IL-4 expression (pN3.Cox2.IL-4) (40). In an equine chondrocyte model, transfection with pN3.Cox-2.IL-4 of IL-1β or LPS-stimulated cells resulted in increased IL-4 expression and decreased expression of the inflammatory cytokines IL-1β,−6,−8 and the matrix degrading-enzymes MMP-1 and−3. Recently, Song et al. investigated the therapeutic potential of IL-4 overexpressing mesenchymal stem cells in spheroids (IL-4 MSC spheroid) (41). MSCs in spheroids are less prone to cell death after IA injection than naïve MSCs. In IL-1βstimulated primary rat chondrocytes *in vitro*, IL-4 MSC spheroids led to a reduction of IL-1β-induced apoptosis and a decrease in the production of OA factors (i.e., NO, iNOS, MMP-13), as well as an increase in the synthesis of cartilage extracellular matrix (ECM) (i.e., Col2). *In vivo*, in a rat anterior cruciate ligament transection (ACLT) with partial medial meniscectomy (MMx) (ACLT-MMx) model, an increased attenuation of tissue regeneration was observed after IA implantation of IL-4 MSC spheroids, along with improved chondroprotective and anti-inflammatory effects and better pain relief. Interestingly, these effects were higher in IL-4 MSC spheroids than in either IL-4 naïve MSCs or MSC spheroids without IL-4 suggesting that IL-4 MSC spheroids may increase the therapeutic efficacy of MSCs.

##### 4.1.1.5. TSG6

Tumor necrosis factor-inducible gene 6 (TSG6) is an HA-binding protein associated with inflammatory processes. However, several studies report its protective effects in experimental arthritis. Inflammation and cartilage damage being two components of OA, Broeren et al. first demonstrated the functionality of TSG6 gene therapy *in vitro* by evaluating its effects on osteoclasts (42). They observed that overexpression of TSG6 using pShuttle-CMV-TSG-6, inhibited the resorption activity of osteoclasts. They then evaluated the effect of TSG6 therapy *in vivo*, in a CIOA mouse model. While no difference in protease activity or cartilage damage was observed, there was an increase in ectopic bone formation. Taken together, these data suggest that IA gene therapy with TSG6 does not seem to be a promising treatment for OA.

##### 4.1.1.6. CrmA

The cytokine response modifier A (CrmA) is an inhibitor of caspases and IL-1β converting enzyme proteases and plays a role in attenuating IL-1β-induced inflammation and apoptosis in OA chondrocytes. Previously, due to their good biocompatibility, biodegradability and high stability, HA/CS microspheres have been shown to be a secure vehicle for the release of drugs. In this context, Qiu et al. investigated the use of these microspheres as vectors to deliver CrmA pDNA into OA synoviocytes (43). They demonstrated a decrease in IL-1β-mediated inflammation *via* a significant reduction of MMPs (MMP-3 and−13) in the HA/CS/pCrmA group compared to the control group.

#### 4.1.2. Growth factors and receptors

In OA pathophysiology, the balance between catabolic and anabolic processes is crucial for the phenotype of chondrocytes. Thus, a promising approach in the context of gene therapy would be to either inhibit degradation or stimulate the synthesis of the ECM. Previously, it has been shown that insulin-like growth factor 1 IGF-1 stimulates matrix synthesis by stimulating type II collagen and aggrecan synthesis and promotes chondrocyte proliferation (Table 2).

**Table 2:** Growth factors and receptors.

**References**	**Objectives**	**Study design**	**Disease models**	**Delivery methods**	**Targets**	**Effects**
Manning et al. (45)	Evaluate co-expression of IGF-1 and IL-4 in an *in vitro* inflammatory model	Preclinical *in vitro*	IL-1β /TNF-α-stimulated canine chondrocytes	pVitro2-IGF-1; pVitro2-IGF-1/IL-4 (transfection with Fugene6)	IGF-1 and IL-4	Reduced pro-inflammatory mediators and IGF-binding proteins; increased type II collagen and proteoglycans
Weimer et al. (46)	Investigate efficient and prolonged IGF-I overexpression *via* rAAV transfection and its effect on restoring OA cartilage	Preclinical *in vitro* and *in situ*	Human OA chondrocyte monolayer cultures and alginate spheres; human OA explant cultures	Adenoassociated virus: rAAV-hIGF-I	IGF-I	Increased proliferation; decreased apoptosis; increased levels of proteoglycan and type II collagen; increased cell proliferation *in situ*; decreased apoptosis *in situ*; increased proteoglycan and type II collagen content *in situ*
Aguilar et al. (47)	Determine efficiency of pAAV/IGF-I transfection of chondrocytes and determine effect of endogenous *vs*. exogenous IGF-I delivery	Preclinical *in vitro*	Mature vs. neonatal articular bovine chondrocyte culture (carpal joints vs. stifle condyles)	Adenoassociated virus: pAAV/IGF-I transfection vs. exogenous IGF-I stimulation	IGF-I	Dose-dependent increase of IGF-I production after transfection with pAAV/IGF-I; mature chondrocytes respond better than neonate chondrocytes; exogenous delivery into cell culture medium showed lower results
Aguilar et al. (48)	Development of new peptide-based material with high affinity to IGF-I	Preclinical *in vitro*	Neonatal articular bovine chondrocyte culture (stifle condyles)	Hydrogels; alginate (transfection with Fugene 6)	IGF-I	Enhanced binding affinity of IGF-I; extended IGF-I availability; increased GAG and HYPRO synthesis
Ko et al. (49)	Evaluate the effects of relaxin expression on fibrosis inhibition in OA synovial fibroblasts	Preclinical *in vitro*	Human OA synovial fibroblasts	Adenovirus: Ad-RLN	Relaxin	Anti-fibrogenic effects on OA synovial fibroblasts *via* inhibition collagen synthesis and collagenolytic pathways such as MMP-1,-13, TIMP-1 and -2
Ulrich-Vinther et al. (50)	Investigate potential of TGF-β1 overexpression to restore cartilage anabolism	Preclinical *in vitro*	Human primary OA chondrocytes	Adenoassociated virus: AAV-TGF-beta1-IRES-eGFP	TGFβ1	Increased expression of type II collagen, aggrecan; decreased expression of MMP3
Venkatesan et al. (51)	Investigate potential of TGF-β1 overexpression to restructure OA cartilage	Preclinical *in vitro* and *in situ*	Human primary OA chondrocytes and OA cartilage explants	Adenoassociated virus: rAAV-hTGF-beta	TGFβ1	Increased cell proliferation; reduced apoptosis; increased proteoglycan and type-II collagen deposition; decreased type-X collagen content; decreased hypertrophic differentiation players (MMP13, PTHrP and beta-catenin); increased protective TIMP-1 and TIMP-3 expression
Noh et al. (52)	Evaluate potential of TGF-β1-secreting human chondrocytes (TG-C) to regenerate cartilage	Preclinical *in vivo*	Rabbit surgically induced single partial cartilage defect model	Retrovirally induced human chondrocytes	TGFβ1	Dose-dependent effect on cartilage regeneration
			Goat surgically induced single full-thickness cartilage defect model	TG-C		Increased proliferation of new chondrocytes; positive effect on joint cartilage at 6 months
Lee et al. (53)	Evaluate the effects of TissueGeneC on pain and cartilage structure *via* the polarization of M2 macrophages	Preclinical *in vivo*	Rat MIA model	TissueGeneC	TGFβ1	Pain relief and cartilage structural improvement; increased IL-10 in the synovial fluid; induction of arginase 1 expression (M2 macrophages marker) and decreased CD86 (M1 macrophages marker) → Polarization of M2 macrophages
Gao et al. (54)	Compare BMP2 delivery by coacervation and lentiviral delivery on cartilage repair	Preclinical *in vitro*	hMDSCs	Lentivirally (LBMP2/GFP) transduced hMDSCs; coacervate sustain release technology	BMP-2	LBMP2/GFP transduction increases chondrogenic differentiation of hMDSCs
		Preclinical *in vivo*	Rat MIA model			hMDSC-LBMP2/GFP improves cartilage repair and of cartilage erosion; coacervate delivery of BMP2 similar articular cartilage regeneration than with hMDSC-LBMP2/GFP
Matsumoto et al. (55)	Evaluate the effect of BMP-4 and Flt-1-transduced MDSCs on cartilage repair in a rat OA model	Preclinical *in vivo*	Rat MIA model	Retrovirally transduced MDSCs	BMP-4 •Flt-1	BMP-4-transduced MDSCs lead to good cartilage repair, but with osteophyte formation; exacerbated effect without osteophyte formation with the combination of sFlt-1 and BMP-4-transduced MDSCs; higher levels of chondrocyte differentiation and proliferation; lower levels of chondrocyte apoptosis
Tang et al. (56)	Assess the effect of follistatin delivery on metabolic inflammation and knee OA caused by a high-fat diet	Preclinical *in vivo*	Mouse DMM model	Adenoassociated virus: AAV9-FST	Follistatin (FST)	Reduced cartilage degeneration; decreased joint synovitis; lower levels of pro-inflammatory cytokines; normalization of obesity-induced increased heat withdrawal latency; enhanced muscle growth and muscle performance; protection from injury-mediated trabecular and cortical bone structure changes
Chen et al. (57)	Evaluate the effects of nanomicrosphere-delivered GDF-5 on OA	Preclinical *in vitro*	Rabbit chondrocytes	Nanomicrospheres	GDF-5	Increased expression of collagen II and aggrecan
		Preclinical *in vivo*	Rabbit ACLT and menisectomy model			Improved cartilage morphology and joint structure

##### 4.1.2.1. IGF-1

Manning et al. evaluated the co-expression of IGF-1 and IL-4 using a dual promoter plasmid pVitro2, in an *in vitro* inflammatory model (45). In canine chondrocytes stimulated with IL-1β or TNF-α, the authors observed a decrease of pro-inflammatory mediators (i.e., IL-1β, TNFα, and IL-6) in the presence of IGF-1/IL-4 (pVitro2-IGF-1/IL-4), contrary to cells transfected with IGF-1 alone (pVitro2-IGF-1). They also observed a decrease in IGF-binding proteins and an increase in the key cartilage matrix proteins type II collagen and proteoglycans. These data suggested that the combination of genes could provide a better perspective in the context of gene therapy. Weimer et al. studied the overexpression of IGF-1 by rAAV transfection (rAAV-hIGF-1) and its effect on OA cartilage restoration in primary human OA chondrocytes *in vitro* and in explant cultures *in situ* (46). Prolonged IGF-1 secretion increased proliferation levels of chondrocytes and decreased apoptosis. Proteoglycan and type II collagen levels were also increased. Aguilar et al. compared the efficacy of AAV-mediated upregulation of IGF-1 (pAAV/IGF-1) (47). In articular bovine chondrocyte cultures and after transfection with pAAV/IGF-1, they observed a dose-dependent increase of IGF-1 and GAG production (58). However, IGF-1 added to media was less effective than endogenously produced IGF-1. In 2017, Aguilar et al. continued their studies on IGF-1 and developed a new material based on peptides (IGFBP-5) with a high affinity for IGF-1 and grafted a binding peptide sequence from IGFBP-5 onto alginate (48). Results demonstrated an increased binding affinity and availability of IGF-I as well as an increased synthesis of GAGs and hydroxyproline. According to the authors, “these data demonstrate the coordinated engineering of cell behavior and material chemistry to greatly enhance extracellular matrix synthesis and tissue assembly and can serve as a template for the enhanced performance of other therapeutic proteins” (48). Taken together, all these studies suggest that IGF-1 is a promising candidate for OA gene therapy.

Besides IGF-1, another target was studied, namely relaxin (RLN) (Table 2). This hormone belonging to the insulin superfamily, down-regulates TGF-β1-mediated collagen production and is important for matrix turnover by regulating MMP expression in the cartilage of synovial joints. In this context, Ko et al., investigated i*n vitro* the effects of Ad-mediated RLN (Ad-RLN) expression on fibrosis inhibition in human OA synovial fibroblasts (49). Compared to control cultures, authors observed in Ad-RLN transfected synoviocytes, an increase of MMP-1 and conversely, a decrease of collagen IV, TIMP-1, and−2 protein expression suggesting that RLN exerts anti-fibrogenic effects on OA synovial fibroblasts *via* inhibition of collagen synthesis and collagenolytic pathways.

Taken together, IGF-1 gene therapy increased the production of major matrix components, such as proteoglycans and type II collagen, while also reducing pro-inflammatory mediators.

##### 4.1.2.2. TGF-β1

The transforming growth factor-β (TGF-β) family has more than 35 members, including TGF-β, activins, and bone morphogenetic proteins (BMPs). Three isoforms of TGF-β are known (TGF-β1, -β2, and -β3), TGF-β1 being amongst the most prevalent growth factors involved in cartilage repair. Indeed, it directly stimulates the synthesis of proteoglycans and collagen, and antagonizes the effects of IL-1 on metalloproteinases in both normal and OA chondrocytes. In this context, Ulrich-Vinther et al. were among the first to evaluate the effect of overexpression of TGF-β1 on cartilage anabolism in human OA chondrocytes (Table 2) (50). They demonstrated that after AAV-TGF-β1 transduction, chondrocytes expressed higher levels of type II collagen and aggrecan, while the expression of MMP-3 was decreased. Venkatesan et al. also investigated the overexpression of TGF-β1 (rAAV-hTGF-beta) in human primary OA chondrocytes and OA cartilage explants (51). They observed an increase in proliferation and cell survival compared to the control vector. They highlighted a decrease of key markers of chondrocyte hypertrophy such as type-X collagen, MMP-13, the parathyroid hormone-related protein **(**PTHrP) and β-catenin and, on the contrary, an increased expression of protective TIMP-1 and−3. The development of TissueGene-C (TG-C), a mix of human allogeneic chondrocytes and irradiated cells overexpressing TGF-β1, represented a key step for the advancement of OA gene therapy. Thus, Noh et al. evaluated the potential of TG-C *in vivo*, in a rabbit surgically induced cartilage defect OA model. In the presence of TG-C, they observed a dose-dependent effect on cartilage regeneration (52). It was also studied in goats in a surgically induced single full-thickness cartilage defect model. They observed an increase in the proliferation of new chondrocytes and a positive effect on articular cartilage at 6 months. After a demonstration of safety and efficacy, the authors proceeded with a phase I clinical study of TG-C (Table 3). Ha et al. evaluated the dose-response of this cell therapy in OA patients (59). This was a single center, open-label, dose escalation study on 12 adults, to assess the dose-response of three different doses of TG-C. No serious treatment-related adverse events were observed. Swelling, effusion, and minor local reactions were dose dependent. Knee evaluation scores (KSCRS, WOMAC, and VAS) seemed to point toward a dose-dependent trend of efficacy. Cherian et al. led the development of a clinical phase II (60). The objective was to evaluate the efficacy of TG-C in patients with grade III OA (*n* = 102). The characteristics of the study were as follows: multi-center, double-blinded, placebo-controlled, and randomized. The authors observed improved knee function and pain as assessed by the International Knee Documentation Committee (IKDC) and the Visual Analog Scale (VAS) and in parallel, reduced analgesics use by patients. A phase IIa study evaluated the efficacy and safety of TG-C in patients who had late-stage knee OA (grade IV) (*n* = 27) (61). This was a multi-center and single-blinded study. There was an improvement in both symptoms and activity level and function of the knee; stiffness, motor function and pain improved significantly, as evidenced by improved IKDC, WOMAC, and VAS scores. Of note, approval of TG-C as gene therapy for OA under the name of *Invossa* was revoked in Korea when it became apparent that the transgenic cells were mainly HEK293 cells instead of chondrocytes, and its status is currently being investigated. In the US, on the other hand, trials are ongoing as HEK293 cells had been used from the beginning [reviewed in (64)].

**Table 3:** Clinical trials.

**References**	**Objectives**	**NCT #**	**Participants**	**Study design**	**Disease models**	**Delivery methods**	**Targets**	**Effects**
Ha C-et al. (59)	Evaluate the dose-response of TissueGeneC in OA patients	NCT02341391	Adults with KL grade IV knee OA	Clinical Phase I, single center, open-label, dose-escalation	Human OA patients	TissueGeneC	TGFβ1	No treatment-related serious adverse events; swelling, effusion and minor reactions are dose-dependent; a dose-dependent trend efficacy
Cherian et al. (60)	Evaluate efficacy of treating grade III OA patients with genetically engineered allogenic human chondrocytes expressing TGF-β1	NCT01221441	Adults with KL grade III knee OA	Clinical phase II: multi-center, double-blinded, placebo-controlled, randomized	Human OA patients	Retrovirally transduced allogenic human chondrocytes (TissueGene-C)	TGFβ1	Improved keen function and pain as assessed by IKDC and VAS; less analgesic use
Ha et al. (61)	Evaluate the efficacy and safety of a cell-mediated therapy in OA patients	NCT02341378	Adults with KL grade IV knee OA	Clinical phase IIa, multicenter, single -blind	Human OA patients	GEC-TGF-β1 (TissueGeneC)	TGFβ1	Improved symptoms, activity levels and knee function; Significant improvement in stiffness, motor function and pain demonstrated *via* the IKDC, WOMAC and VAS scores
Kim et al. (62)	Evaluate the efficacy and safety of a cell-mediated therapy in OA patients	NCT02072070	Adults with KL grade III knee OA	Clinical Phase III, multicenter, double-blind	Human OA patients	TissueGeneC	TGFβ1	Decreased serum CTX-1 and urine CTX-II levels over 1 year in TG-C than placebo-treated patients; significant improvements in function and pain in OA patients; frequent adverse events were edema, arthralgia, joint swelling and injection site pain
NA	Post Marketing Surveillance on Safety and Effectiveness Evaluation of INVOSSA	NCT03412864	Adults with KL grade III knee OA	Post marketing surveillance study	Human OA patients	INVOSSA	TGFβ1	No outcome data
NA	Preliminary Evaluation of Safety, Tolerability, and Efficacy of XT-150	NCT03282149 NCT03477487 NCT03769662	Adults for whom replacement knee surgery is recommended	Clinical Phase I, single center, dose escalation	Human OA patients	hIL-10var pDNA (XT-150)	IL-10	No outcome data
NA	Evaluate the efficacy and safety of XT-150 in patients experiencing moderate to severe pain due to OA of the knee	NCT04124042	Adults with WOMAC pain score ≥ 8	Clinical phase II: multi-center, double-blinded, placebo-controlled, randomized	Human OA patients	hIL-10var pDNA (XT-150)	IL-10	No outcome data
Sellon et al. (63) (reviewed by Evans C 2022) (64)	Evaluate safety of three different doses of sc-rAAV2.5IL-1Ra	NCT02790723	Adults with moderate OA of the knee	Clinical Phase I, single center, open-label, dose-escalation	Human OA patients	Sc-rAAV2.5IL-1Ra	IL-1Ra	No outcome data
Kelley et al. (65) (reviewed by Evans C 2022) (64)	Evaluate the Safety and Tolerability of FX201	NCT04119687	Adults with KL grade II, III or IV knee OA	Clinical Phase I, single center, open-label, dose-escalation	Human OA patients	HDAd-IL-1Ra (FX201)	IL-1Ra	No outcome data

Thus, Kim et al. conducted a phase III (NCT03291470), multi-center and double-blinded clinical trial including a total of 163 Kellgren–Lawrence grade III patients (62). Results highlighted a decrease in serum CTX-I and urine CTX-II levels, as well as significant improvements in function and pain in OA patients. Recently, Lee et al. investigated the effects of TG-C on pain and cartilage structure *via* the polarization of M2 macrophages (53). In an OA rat monosodium iodoacetate (MIA) model, pain relief and improvement of cartilage structure were demonstrated; an increase in IL-10 and TGF-β1 in synovial fluid was observed followed by induction of arginase 1 expression (M2 macrophage marker) and decrease of CD86 (M1 macrophage marker). The effect was therefore favorable for the induction of M2 macrophages implicated in tissue repair.

In summary, TGF-β1 gene therapy mainly restored synthesis of proteoglycan and type II collagen, while also reducing markers of hypertrophic differentiation.

##### 4.1.2.3. BMPs

Bone morphogenic proteins (BMPs) are important players in the formation of functional joints and in the maintenance of cartilage homeostasis. Both BMP2 and BMP4 are involved in chondrogenesis, cartilage growth, and chondrocyte proliferation (23) and are therefore very interesting targets for OA gene therapy (Table 2). Muscle-derived stem cells (MDSCs) are a promising cell type for tissue engineering to allowre musculoskeletal regeneration. Gao et al. showed that hMDSCs lentivirally transduced to overexpress BMP2 have an increased chondrogenic differentiation capacity (54). In addition, these lentivirally-transduced hMDSCs improved cartilage repair and erosion in a rat MIA model *in vivo*. Interestingly, BMP2 delivery by coacervation resulted in similar articular cartilage repair as lenti-BMP2/GFP-mediated delivery. Matsumoto et al. showed that hMDSCs retrovirally transduced to overexpress BMP4 and transplanted into a rat MIA model of OA, are able to repair articular cartilage; osteophyte formation was however observed in some areas of the joint (55). A combination of soluble Fms-like Tyrosine Kinase 1 (sFLT-1) and BMP4-transduced hMDSCs exacerbated this effect without osteocyte formation. Moreover, increased differentiation and proliferation and decreased apoptosis was observed in chondrocytes in the animals transplanted with sFLT-1 and BMP4-transduced hMDSCs.

##### 4.1.2.4. FST

Recently, Tang et al. were interested in the effect of follistatin (FST) delivery on metabolic inflammation (Table 2) (56). In the OA pathology, the authors assume that “FST delivery using a gene therapy approach has multifactorial therapeutic potential through its influence on muscle growth via inhibition of myostatin activity as well as other members of the TGFβ family” (56). In a mouse destabilization of the medial meniscus (DMM) OA model, FST gene therapy (AAV9-FST) reduced cartilage degeneration and decreased joint synovitis. The overexpression decreased the inflammatory cytokine, IL-1β and restored muscle performance by enhancing muscle growth and muscle performance. Finally, this therapy protected injury-mediated trabecular and cortical bone structure changes. This systemically delivered therapy is very promising especially for OA, but also for associated metabolic conditions, such as diseases of muscle wasting.

##### 4.1.2.5. GDF-5

Like Deng et al. for IL-Ra, Chen et al. investigated a non-viral gene therapy vector to evaluate the therapeutic potential of the growth and differentiation factor-5 (GDF-5) (Table 2) (57). This factor belongs to the TGF-β and BMP superfamilies that regulate cell growth and differentiation. They generated nano-microspheres (NMPs) consisting of chitosan, HA, and chondroitin sulfate, and containing a GDF-5 plasmid. In rabbit chondrocytes, these GDF-5 containing NMPs increased the expression of collagen II and aggrecan, two key ECM proteins. In a rabbit ACLT and meniscectomy OA model, the authors demonstrated that these NMPs improved cartilage morphology and joint structure compared to the saline control group.

#### 4.1.3. Transcription factors

Targeting transcription factors for OA gene therapy, such as sex-determining region Y-type high mobility group box 9 (SOX9), krüppel-like factor 2 or 4 (KLF2/KLF4), or activating transcription factor 4 (ATF-4), has the benefit of correcting gene expression profiles altered in OA toward the expression of genes implicated in the production of ECM compounds.

##### 4.1.3.1. SOX9

SOX 9 is of particular interest because of its role in cartilage formation and chondrocyte differentiation. Furthermore, its expression is notably downregulated in OA (Table 4). The combinatorial effect of SOX9 and FGF2 overexpression after rAAV-mediated gene delivery to human OA chondrocytes or OA cartilage explants was evaluated by Cucchiarini et al. (66). They observed a higher production of proteoglycans and type II collagen, which was attributed to SOX9 rather than FGF2. The same effects were observed by Daniels et al. when transforming human articular OA chondrocytes in a 3D aggregate culture model after rAAV-mediated SOX9 overexpression (67). The combinatorial overexpression of SOX9 and TGF-β1 after rAAV-mediated gene delivery to human OA chondrocytes or OA cartilage explants as evaluated by Tao et al. showed the same effects, along with increased proliferation and cell density and enhanced levels of chondrogenic aggrecan (ACAN) and collagen type II alpha 1 chain (COL2A1) expression (68). The downside of rAAV-mediated gene delivery is however the widespread presence of neutralizing antibodies against the viral proteins in the host. Therefore, Urich et al. explored the potential of delivering rAAV-FLAG-hsox9 vectors *via* polymeric micelles into human primary OA chondrocytes. These micellar systems increased proliferation, type II collagen, and proteoglycan deposition in human primary OA chondrocytes stimulated with IL-1β and TNF-α (69).

**Table 4:** Transcription factors.

**References**	**Objectives**	**Study design**	**Disease models**	**Delivery methods**	**Targets**	**Effects**
Cucchiarini et al. (66)	Evaluate the combinatorial effect of FGF-2 and Sox9 *via* rAAV gene transfer upon the OA cartilage	Preclinical *in vitro* and *in situ*	Human articular OA chondrocytes (3D culture model) and OA cartilage explants	Adenoassociated virus: rAAV-hFGF-2; rAAV-FLAG-h*sox9*	FGF-2 SOX9	Improvement both survival and proliferation of chondrocytes with single overexpression of FGF-2; Combination of FGF-2 and Sox-9 ↑production of proteoglycans and type-II collagen, ↓ expression of type-X collagen
Daniels et al. (67)	Evaluate the effects of rAAV-mediated sox9 overexpression on the biological activities of human OA chondrocytes	Preclinical *in vitro*	Human articular OA chondrocytes (3D culture model)	Adenoassociated virus: rAAV-FLAG-h*sox9*	SOX9	Significant production of major matrix components (proteoglycans and type-II collagen)
Tao et al. (68)	Assess the effect of TGF-beta and SOX9 co-overexpression	Preclinical *in vitro* and *in situ*	Human OA chondrocyte monolayer cultures and alginate spheres; human OA explant cultures	Adenoassociated virus: rAAV-hTGF-beta; rAAV-FLAG-hsox9	TGF-beta SOX9	Increased proliferation and cell density; enhanced deposition of matrix proteoglycans; enhanced type-II collagen deposition; enhanced levels of chondrogenic SOX9, ACAN and COL2A1 expression; reduced type-X-collagen expression
Urich et al. (69)	Investigate the ability of polymeric micelles to deliver therapeutic rAAV-FLAG-hsox9 into human OA chondrocytes	Preclinical *in vitro*	Human primary OA chondrocytes in presence of pro-inflammatory cytokines	Adenoassociated virus: rAAV-FLAG-hsox9/Polymerci micelles	SOX9	Increased type-II collagen deposition; increased proliferation and proteoglycan deposition
Kawata et al. (70)	Evaluate the therapeutic effects of KLF4 and KLF2 for OA	Preclinical *in vitro*	Human OA chondrocytes, meniscal cells and BMSCs	Adenovirus: Ad-KLF2 and Ad-KLF4	KLF2 and KLF4	Upregulation of cartilage genes
		Preclinical *in vivo*	Mouse DMM model	Adenoassociated virus: AAV-KLF4	KLF4	Upregulation of cartilage genes; improved pain; improved OARSI score, meniscus histopathological score, synovitis score and bone score
Wang et al. (71)	Explore therapeutic effects of serum-derived exosomes from OA mice	Preclinical *in vivo*	Mouse anterior medial meniscus excision OA model	OA exosomes: ATF4-OA-Exo	ATF4	Alleviated cartilage damage of OA mice (proteoglycan loss, increased Mankin score and increased osteophytes); increased COL-II and decreased MMP13 and inflammatory cytokines; partial recovery of weakened autophagy; inhibited TM/TNF-alpha induced chondrocyte apoptosis

##### 4.1.3.2. KLF2 and 4

Interestingly, OA is associated with a decreased expression of KLF family members of transcription factors (72). Kawata et al. evaluated the therapeutic potential of KLF2 and 4 for OA. In human OA chondrocytes, meniscal cells or BMSCs, Ad-mediated overexpression of KLF2 or 4 induced an upregulation of cartilage genes, including COL2A1, COL11A2, COMP, PRG4, and SOX9 (70). Furthermore, AAV-mediated overexpression of KLF4 in a mouse DMM OA model not only upregulated above mentioned cartilage genes, but also resulted in reduced pain and an improved OARSI score, meniscus histopathological score, synovitis score, and bone score. The authors concluded “that KLF4 had therapeutic and protective effects against OA-associated tissue damage and pain” (70).

##### 4.1.3.3. ATF4

Exosomes are small extracellular vesicles, which can contain proteins, RNA, and DNA, making them very interesting vehicles for gene delivery because of their high penetration and low immunogenicity. Wang et al. were the first to explore the therapeutic effects of serum-derived exosomes from meniscus-injury-induced OA mice overexpressing ATF4 (ATF4-OA-Exo) in a meniscus injury-induced mouse model of OA (Table 4) (71). These ATF-OA exosomes alleviated the cartilage damage and proteoglycan loss observed in these OA mice and decreased the number and size of osteophytes, as well as the cartilage Mankin score. In line with these observations, type II collagen was increased, while MMP-13 and inflammatory cytokines were decreased. In addition, ATF4-OA-exosome injection led to a partial recovery of the weakened autophagy in OA cartilage and inhibited apoptosis in tunicamycin (TM)- or TNF-α-treated chondrocytes, indicating that the effect ATF4-OA-Exo might be exhibited through the induction of autophagy.

#### 4.1.4. Other key targets

##### 4.1.4.1. PRG4

Proteoglycan 4 (PRG4), also known as lubricin, is a glycoprotein expressed by chondrocytes and synoviocytes and naturally secreted in synovial fluid. It is of particular interest for gene therapy, as it acts as a lubricant to reduce friction within articular cartilage, diminishes inflammation, and as an anabolic factor slows down OA progression (Table 5). In humans, Camptodactyly-Arthropathy-Coxa Vara-Pericarditis Syndrome, characterized by early onset OA, is caused by loss-of-function mutations in PRG4. Similarly, PRG4 knock-out mice also develop early OA. Ruan et al. reported that transgenic, joint-specific PRG4 overexpression protects mice against OA development and motor impairments in a mouse ACLT model and protects aging mice from natural OA development (73). Treatment with an HDV vector expressing PRG4 (HDV-PRG4) protected mice against OA development and improved structural joint status in a mouse ACLT model, as evidenced by improved histology scores, cartilage volume and bone area covered by cartilage. Later, Ruan et al. reported that in the mouse ACLT model, IA injection of HDV-PRG4 or a capsid-modified HDV (a10mabHDV-PRG4, which specifically targets chondrocytes), prior to surgical OA induction, protected animals from OA development (74). Cartilage volume and bone area covered by cartilage were preserved in both cases. The efficacy was, however, greater for the a10mabHDV-PRG4. When injecting viral vector 2 weeks after ACLT surgery, a 10-times lower effective dosage of a10mabHDV-PRG4 was needed to prevent post-ACTL OA compared with the untargeted HDV-PRG4. Using the low dose of a10mabHDV-PRG4, the preservation of cartilage volume was greater, and it covered a larger area of bone compared to the same dose of HDV-PRG4 vector. The effect of a10mabHDV-PRG4 was similar to the 10-fold higher dose of HDV-PRG4. Stone et al. explored the added benefits of a combinatorial gene therapy approach compared to targeting PRG4 alone (75). The IA co-injection of HDVs expressing IL-1Ra and PRG4 in a mouse ACLT model, resulted in better preservation of cartilage volume and bone area covered by cartilage than injection of either HDV alone. This was accompanied by the increase of anabolic and the decrease of catabolic and inflammatory gene expression. In the less severe DMM model, both PRG4 and IL-1Ra + PRG4 combinatorial gene therapy were able to maintain cartilage volume and covered surface area. While all treatment groups prevented OA-induced thermal hyperalgesia at the early timepoint, only the combined gene therapy showed significant protective effect at the late timepoint. The chondroprotective effect of PRG4 gene therapy was confirmed by Seol et al. in a rabbit ACLT model (76). IA injection of AAV-PRG4-GFP immediately after ACLT surgery inhibited cartilage damage and reduced the severity of post-traumatic OA (PTOA). In addition, more cartilage surface and superficial chondrocytes were covered by lubricin.

**Table 5:** Other targets.

**References**	**Objectives**	**Study design**	**Disease models**	**Delivery methods**	**Targets**	**Effects**
Ruan et al. (73)	Evaluate functionality of HDV-PRG4 in OA mouse model	Preclinical *in vivo*	Mouse ACLT model	HDV-PRG4	PRG4	Improved histology scores, cartilage volume and coverage
Ruan et al. (74)	Development of a targeted vector for chondrocyte-specific delivery of target genes for OA therapy	Preclinical *in vivo*	Mouse ACLT model	(Modified) helper-dependent adenovirus: (a10mab)HDV-PRG4	PRG4	Prevention of OA development with early treatment with both HDV-PRG4 and a10mabHDV-PRG4; greater efficacy of a10mabHDV-PRG4; preserved cartilage volume and surface area; with late treatment, greater preservation of cartilage volume; larger bone area covered by cartilage with a10mabHDV-PRG4 compared to HDV-PRG4 vector, resulting in 10-fold reduction of effective dosage requirement for preventing post-ACTL OA
Stone et al. (75)	Evaluate the beneficial effects of a combinatorial gene therapy approach compared to monotherapy	Preclinical *in vivo*	Mouse DMM and mouse ACLT model	Helper-dependent adenovirus: HDV-NFκB-IL1ra and HDV-EF1-PRG4	IL-1Ra and PRG4	ACLT model: better preservation of cartilage volume and covered surface area in combined therapy; prevented decrease of anabolic gene expression and upregulation of inflammatory and catabolic pathways; DMM model: maintained cartilage volume and covered surface area of underlying bone in combined and PRG4 therapy; longer prevention of OA-induced thermal hyperalgesia with combined therapy
Seol et al. (76)	Evaluate functionality of recombinant PRG4-GFP fusion protein in delaying OA progression	Preclinical *in vivo*	Rabbit ACLT model	Adenoassociated virus: AAV-PRG4-GFP	PRG4	Reduced post-ACLT severity of PTOA; higher percentage of cartilage surface and superficial chondrocytes coated with lubricin
Tashkandi et al. (77)	Assess the potential of LOXL2 to be used for translational research and clinical applications in OA treatment	Preclinical *in vitro*	IL-1β stimulated ATDC5 cartilage cell line	Adenovirus: Adv-RFP-LOXL2	LOXL2	Blunted decrease of Acan and Sox9; attenuated expression of Adamts4/5 and MMP13; attenuated IL-1β induced NF-κB activity
		Preclinical *in vivo*	Chondrodysplasia (Cho/+) mice			Protection against progressive OA: increased proteoglycan deposition; increased expression of aggrecan and Col2; decreased expression of Mmp13 and Adamts5; increased expression of anabolic genes
			MIA-induced LOXL2 transgenic mice	LOXL2 transgenic mice (loxP-PGK-neo-stop-loxP-LOXL2-IRES-eGFP)		Protection against MIA-induced proteoglycan and aggrecan degradation and decreased Mmp13 expression; protection against MIA-induced OA-related decline in knee function
Venkatesan et al. (78)	Develop a non-viral gene transfer strategy to stimulate GAG synthesis to promote cartilage repair	Preclinical *in vitro*	IL-1β stimulated primary rat chondrocytes; IL-1β stimulated cartilage explants	Transfection of pShuttle-GlcAT-I using PEI	GlcAT-I	Inhibited IL-1β induced loss of PGs; increased GAG content but no influence on chain size; increased amount of CS chains; restored PG synthesis in IL-1β treated cartilage explants
Fu et al. (79)	Explore the effect of GGCX overexpression on ACLT-induced OA	Preclinical *in vivo*	Rabbit ACLT model	Lentivirus GGXC	GGCX	Reduced morphological changes caused by ACLT; increased cMPG to normal levels; decreased ACLT-induced inflammation (expression of TNFα and IL-1β); decreased collagen type X and MMP13 expression, increased collagen type II expression
Hsieh et al. (80)	Evaluate the effects of Ad-mediated kallistatin overexpression in OA rat model	Preclinical *in vivo*	Rat ACLT model	Adenovirus: AdHKBP	Kallistatin	Reduced inflammatory response (IL-1β and TNF-α levels in joints); reduced OA severity and apoptosis; decreased macrophage infiltration; reduced hyperplasia and synovitis
Ashraf et al. (81)	Determine effect of Rheb on phenotype and function of OA chondrocytes	Preclinical *in vitro*	Human articular OA chondrocytes	Transfection of pEGFP-N1 vector using microporator	RHEB	Normalized morphology; reduced senescence; decreased oxidative stress
	Determine effect of Rheb expression on OA progression in mice	Preclinical *in vivo*	Mouse DMM model	Adenovirus: Ad-Rheb		Attenuated cartilage destruction; suppressed expression of Adamts5, Mmp13, Col10 and Col2a1; inhibited apoptosis
Grossin et al. (82)	Determine efficiency of gene transfer with HSP70 in rat patellar cartilage	Preclinical *in vivo*	Rat MIA model	Transfection of pcDNA3.1/CT-GFP-HSP70 by electroporation	HSP70	Inhibited endochondral ossification in the deep layer; reduced severity of OA-induced lesions
Yoon et al. (83)	Identify the role of PUM1 in OA progression	Preclinical *in vivo*	Mouse DMM model	Lentivirus: pLenti-GII-CMV-PUM1	PUM1	Reduced cartilage destruction; less chondrocyte loss; reduced OARSI score
Na et al. (84)	Asses the therapeutic potential of sCCR2 E3 for OA	Preclinical *in vivo*	Rat MIA model	sCCR2 E3 vector *via* electroporation	sCCR2 E3	Reduced pain; less bone loss and cartilage degradation; lower OARSI and Mankin score; inhibition of IL-1β, IL-6 and MMP-13 expression
Cao et al. (85)	Elucidate the role of cholesterol-LRP3 axis in OA	Preclinical *in vitro*	TNFa-induced rat OA chondrocytes	Lentivirus: Lv-Lrp3	LRP3	Increased expression of anabolic genes COL2A1, ACAN, SOX9; increased proteoglycan and GAG
		Preclinical *in vivo*	Rat ACLT model			Less cartilage degradation; rescued proteoglycan and type II collagen level; milder OA phenotype; increased expression of anabolic genes COL2A1, ACAN, SOX9; decreased expression of catabolic genes Adamts5 and Mmp13

##### 4.1.4.2. LOXL2

The copper-dependent amine oxidase lysis oxidase-like 2 (LOXL2) catalyzes the first step in the formation of crosslinks in collagens and elastin and has been shown to mediate endochondral ossification. Tashkandi et al. wanted to assess the potential of Ad-mediated overexpression of LOXL2 for OA treatment (Table 5) (77). In an IL-1β stimulated ATDC5 cartilage cell line, LOXL2 overexpression blunted the decrease of aggrecan and SOX9, and attenuated both the IL-1β mediated expression of ADAMTS4/5, and MMP-13, as well as the IL-1β induced activity of NF-κB. In chondrodysplasia (Cho/+) mice, LOXL2 overexpression protected these mice against progressive OA, as evidenced by increased proteoglycan deposition, increased aggrecan, type II collagen, and anabolic gene expression, and decreased MMP-13 and ADAMTS5 expression. Finally, in MIA-induced LOXL2 transgenic mice, LOXL2 overexpression protected against proteoglycan and aggrecan degradation and reduced MMP-13 levels. It protected these mice from MIA-induced OA-related decline in knee function. The authors, therefore, conclude that LOXL2 is a promising target for gene therapy for knee OA.

##### 4.1.4.3. Proteoglycans

Proteoglycans (PG) are made of “core proteins” with covalently attached glycosaminoglycan (GAG) chains. Their depletion is a major hallmark in joint destruction, and factors that might accelerate PG synthesis and deposition are therefore of great interest for gene therapy. Venkatesan et al. investigated how GAG-synthesizing enzyme β1,3-glucuronosyltransferase-I (GlcAT-I) gene therapy might influence cartilage repair in IL-1β stimulated primary rat chondrocytes *in vitro* (Table 5) (78). Lipid-mediated overexpression of GlcAT-I inhibited IL-1β induced loss of PGs, and increased the GAG content and number of chains, rather than chain size. In addition, it restored PG synthesis in IL-1β treated cartilage explants.

##### 4.1.4.4. γ*-Glutamyl Carboxylase*

γ-glutamyl carboxylase (GGCX) regulates the carboxylation of cartilage matrix Gla protein (MPG), a calcification inhibitor. Since the level of uncarboxylated, non-functional MPG (ucMPG) seems to be elevated, while GGCX seems to be reduced in OA patients, Fu et al. set out to investigate the effect of lentiviral-mediated GGCX overexpression in a rabbit ACLT model of OA (Table 5) (79). GGCX overexpression resulted in less ACLT-induced morphological changes and inflammation, which was accompanied by normalized levels of carboxylated MPG. MMP-13 and type X collagen were decreased, while type II collagen was increased to normal levels. GGCX, therefore, constitutes an interesting target for OA gene therapy.

##### 4.1.4.5. Kallistatin

Kallistatin, a serine proteinase inhibitor and known inhibitor of angiogenesis, has been shown to protect cardiomyocytes from apoptosis and to prevent an inflammatory response after myocardial ischemia-reperfusion injury. Hsieh et al. investigated the effect of its Ad-mediated overexpression in a rat ACLT OA model after IA delivery (Table 5) (80). They observed a reduced inflammatory response as evidenced by reduced levels of IL-1β and TNF-α in the joints, as well as an overall reduced OA severity and a reduced number of apoptotic cells. In addition, there was less macrophage infiltration, hyperplasia, and synovitis in Adenoviral vector encoding human kallistatin (AdHKBP) animals, which was exacerbated when treated in combination with HA. Kallistatin gene therapy might therefore be an interesting alternative for OA treatment, especially when administered in combination with HA.

##### 4.1.4.6. RHEB

Ras homolog enriched in the brain (RHEB) is part of the Ras family and is involved in cell growth, proliferation, and differentiation. Ashraf et al. were interested in its effect on OA chondrocytes *in vitro* and OA progression in a mouse DMM model (Table 5) (81). Human articular OA chondrocytes displayed normalized morphology, reduced senescence, and decreased oxidative stress when transfected with a vector containing RHEB cDNA. Ad-mediated overexpression of RHEB in a DMM mouse model resulted in attenuated cartilage destruction, suppressed expression of ADAMTS5, MMP-13, type 10 collagen, and COL2A1, and inhibited apoptosis. The authors, therefore, concluded that RHEB is important for maintaining the chondrogenic phenotype of chondrocytes and is likely involved in preventing the progression of OA *in vivo*.

##### 4.1.4.7. HSP70

Heat shock protein 70 (HSP70) is expressed as a protective agent upon various types of stresses. Grossin et al. set out to investigate its cyto- and/or chondroprotective role in a rat MIA OA model (Table 5) (82). HSP70 overexpression was successfully achieved by electroporation and resulted in the inhibition of endochondral ossification in the deep layer and reduced severity of OA-induced lesions. The authors conclude that HSP70 might therefore be a novel chondroprotective target for gene therapy in OA.

##### 4.1.4.8. PUM1

RNA-binding proteins (RBPs) have been shown to play a role in age-related degenerative diseases (86). Yoon et al. investigated the effect of lentiviral-mediated overexpression of the RBP Pumilio1 (PUM1) in a mouse DMM OA model (83). They observed that it prevented cartilage destruction and chondrocyte loss, and significantly improved the OARSI score. They therefore conclude that PUM1 might be an interesting target for OA therapy.

##### 4.1.4.9. sCCR2 E3

The monocyte chemoattractant protein-1 (MCP-1/CCL2) - C-C chemokine receptor type 2 (CCR2) pathway is involved in OA progression, and ways to block this pathway are being explored (87). Na et al. investigated the effect of a fusion protein constituted of 20 amino acids of the third extracellular domain (E3) of the CCL2 receptor and a soluble CCL2 receptor (sCCR2) in a rat MIA OA model (84). Intra-articular injection of a sCCR2 E3 vector *via* electroporation resulted in reduced pain and less bone loss and cartilage degradation, as well as a lower OARSI and Mankin score. In addition, the expression of inflammatory cytokines IL-1β and IL-6 and catabolic factor MMP-13 was reduced. sCCR2-E3 therefore protects against cartilage damage and inhibits catabolic factors.

##### 4.1.4.10. LRP3

Low-density lipoprotein (LDL) receptor-related proteins (LRPs) play an important part in the regulation of cholesterol metabolism and have recently been shown to be involved in the onset and progression of OA (88). Cao et al. investigated the potential role of LRP3 in OA pathogenesis and therapy (85). In TNF-α stimulated rat OA chondrocytes, lentiviral-mediated overexpression of Lrp3 induced the expression of the anabolic genes COL2A1, ACAN, and SOX9, and restored expression of proteoglycan and GAG. Furthermore, in a rat ACLT OA model, it inhibited cartilage degradation and restored proteoglycan and type II collagen levels. The expression of anabolic genes COL2A1, ACAN, and SOX9 was increased, while the expression of catabolic genes ADAMTS5 and MMP13 was decreased. The authors conclude that LRP3 overexpression may be a new therapeutic target for OA therapy by delaying the degeneration of cartilage.

## 5. Limitations

This scoping review took a rigorous approach based on the JBI framework to the conduct of scoping reviews and was reported following the PRISMA-ScR checklist. To our knowledge, it is the first scoping review of preclinical and clinical studies reporting the effect of gene therapies used to treat OA. It does however suffer from one minor limitation: unpublished work, and ongoing clinical trials were not included. We searched for ongoing clinical trials on gene therapy in OA on clinicaltrials.gov and discussed the outcome of our search in the conclusion and perspective section.

## 6. Conclusions

In the last decade, research on gene therapy has been in full expansion, as evidenced by an increasing number of scientific articles on the subject. In the absence of any DMOAD, gene therapy is of special interest in the context of OA. Both viral and non-viral gene therapy approaches are highly promising treatment alternatives of OA in the future, and several targets have been explored and studied. Both technologies have, however, their own advantages and inconveniences. While viral gene therapy is highly efficient and allows for targeted and sustained delivery of genes, it might activate the innate immune system and cause local inflammation in the host. This is why more recently, several groups have started to explore the option of non-viral gene therapy approaches, such as NMPs or exosomes. While non-viral therapies have the handicap of relatively low transfection efficiency and transient gene expression, they are considered a safer option particularly for OA because of their reduced risk to cause an inflammatory response. CRISPR/Cas9-mediated gene editing, while not the subject of this review, is another alternative for the development of new gene therapies for OA. The technology has revolutionized the field of gene editing and is widely considered an accurate and easy-to-use tool for genome editing. As an example, intra-articular injection of AAV expressing CRISPR/Cas9 components that target MMP-13, IL-1β, and/or nerve growth factor (NGF) suggest that combined deletion of these genes has beneficial effects on both pain management and joint integrity (89). The disadvantage of CRISPR/Cas9 is however the difficulty to deliver enough material to mature cells for efficient genome editing activity. Moreover, it requires the presence of a PAM sequence near the target site, thus limiting its effective targeting range. Most importantly, the relatively high frequency of off-target effects is a significant challenge for employing CRISPR/Cas9 for gene therapy, particularly in clinical applications.

Alternatively, several groups have explored the possibility of targeting multiple genes, so as to heighten and potentiate their effect. In OA with its complex physiopathology, targeting only one actor might not be sufficient to efficiently treat the disease long-term. This strategy seems very promising, and good pre-clinical results have been obtained for various combinations, for example, IL-1Ra and PRG4, FGF2 and SOX9, or IL1-Ra and IL-10.

**Figure 3:**
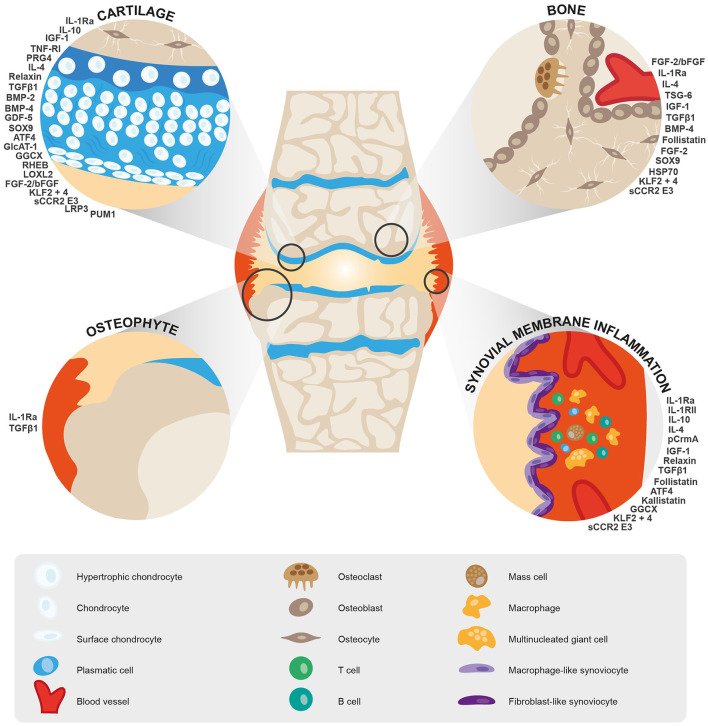
Schematic representation of targets identified for gene therapy of OA and their therapeutic potential.

The main targets identified for gene therapy of OA belong to the interleukin family (IL-1Ra, IL-10, IL-4, TSG6, and CrmA), growth factors and their receptors (IGF-1, relaxin, TGF-β1, BMP2 and 4, follistatin, GDF-5, and FGF2/bFGF), and transcription factors (SOX9, KLF2 and 4, and ATF-4). In addition, lubricin, LOXL2 and other enzymes and proteins are being explored for their therapeutic potential (Figure 3).

Three Phase I studies (NCT03282149, NCT03477487, and NCT03769662) have been successfully completed for XT-150, the locally injectable non-viral therapy expressing interleukin (IL)-10v described by Watkins et al. and discussed above (39). A multinational, double-blind, placebo-controlled Phase II study (NCT04124042) is currently evaluating the safety and efficacy of XT-150 in adults with moderate to severe pain due to knee OA. A Phase I, open-label study (NCT02790723) evaluating the safety of intra-articular injection of scAAV2.5IL-1Ra in nine subjects with moderate knee OA has recently been completed with encouraging results. A Phase I open-label, single ascending dose study (NCT04119687) assessing the safety and tolerability of FX201, the HDAd-IL-1Ra intra-articular gene therapy described by Senter et al., in 72 subjects with moderate to severe knee OA has been recently completed, and the sponsor reported “very compelling” results (no further details were given) (20). A post marketing surveillance study (NCT03412864) including 3,000 OA patients (Kellgren–Lawrence grade III) assessing adverse events as primary endpoints is currently underway for TG-C.

In conclusion, gene therapy is a highly promising treatment for OA, even though it still is in its early stages and further development is required to bring more targets to the clinical stage, as has been done with TG-C targeting TGF-β1, XT-150 targeting IL-10, FX201 and scAAV2.5-IL-1Ra. Overall, the optimal system (s) for safe and effective disease-specific therapy still need to be identified, including the vector type or non-viral delivery system, the therapeutic single gene or gene combination, and the levels of therapeutic gene expression. As more and more clinical trials for gene therapy in OA are being undertaken, we need guidelines for their design and conduct.

## Data availability statement

The original contributions presented in the study are included in the article/Supplementary material, further inquiries can be directed to the corresponding author.

## Author contributions

MU, CL, and YH: drafting manuscript. MU, CL, JG, KG, SP, and YH: revising manuscript content and approving final version of manuscript. All authors contributed to the article and approved the submitted version.

## Glossary

OA, osteoarthritis; DMOAD, disease-modifying osteoarthritis drug; IA, intra-articular; Ad, Adenovirus; HDAd or HDV, helper-dependent adenovirus; AAV, adeno-associated virus; RV, retroviruses; LV, lentivirus; MSCs, mesenchymal stem cells; miRNAs, microRNAs; siRNA, small interfering RNA; circRNA, circular RNA; IL, interleukin; IL-1Ra, interleukin 1 receptor antagonist; ACLT, anterior cruciate ligament transection; IND, investigational new drug; CS, chitosan; HA, hyaluronic acid; pDNA, plasmidDNA; Sc AAV, self-complementary AAV; Nab, neutralizing antibodies; TNF-α, Tumor Necrosis Factor alpha; MMPs, matrix metalloproteinases; bFGF, basic fibroblast growth factor; IGF-1, insulin-like growth factor-1; TIMP-1, Tissue inhibitor matrix metalloproteinase 1; ADAMTS5, disintegrin and metalloproteinase with thrombospondin motifs 5; GAG, glycosaminoglycan; TGFβ1, transforming growth factor-β1; sTNF-RI, soluble tumor necrosis factor-α receptor type I; IL-1RII, type II IL-1β receptor; NO, nitric oxide; iNOS, inducible nitric oxide synthase; PGE_2_, prostaglandin E2; CIOA, collagenase induced osteoarthritis; BM-MSCs, Bone marrow-derived mesenchymal stromal cells; Cox-2, cyclooxygenase-2; LPS, lipopolysaccharide; ECM, extracellular matrix; TSG6, tumor necrosis factor α-stimulated gene 6; CrmA, cytokine response modifier A; IKDC, International Knee Documentation Committee; RLN, relaxin; TIMP-1, tissue inhibitor of metalloprotease-1; BMPs, bone morphogenetic proteins; LAP, pro-peptide latency-associated peptide; LTBPs, latent TGF-β binding proteins;; PTHrP, parathyroid hormone-related peptide; TG-C, TissueGene-C; FDA, Food and Drug Administration; KSCRS, Knee Society Clinical Rating System; VAS, Visual Analog Scale; IKDC, International Knee Documentation Committee; MIA, monosodium iodoacetate; MDSCs, muscle-derived stem cells; sFLT-1, soluble Fms-like Tyrosine Kinase 1; FST, follistatin; DMM, destabilization of the medial meniscus; GDF-5, growth and differentiation factor-5; NMPs, nano-microspheres; SOX9, sex-determining region Y-type high mobility group box 9; KLF, krüppel-like factor; ATF-4, activating transcription factor 4; ACAN, aggrecan; COL2A1, collagen type II alpha 1 chain; RNA, ribonucleic acid; DNA, deoxyribonucleic acid; PRG4, Proteoglycan 4; PTOA, post-traumatic OA; LOXL2, the copper-dependent amine oxidase lysis oxidase-like 2; PG, Proteoglycans; GlcAT-I, GAG-synthesizing enzyme β1,3-glucuronosyltransferase-I; GGCX, γ-glutamyl carboxylase; MPG, matrix Gla protein; ucMPG, non-functional MPG; RHEB, Ras homolog enriched in the brain; HSP70, Heat shock protein 70; RBP, RNA-binding protein; PUM1, Pumilio 1; MCP-1, monocyte chemoattractant protein-1; CCR2, C-C chemokine receptor type 2; LDL, Low-density lipoprotein; LRO, LDL receptor-related proteins; PRISMA-ScR, PRISMA Extension for Scoping Reviews; NGF, nerve growth factor.
