# Supplementary material for: Interleukins, growth factors, and transcription factors are key targets for gene therapy in osteoarthritis: A scoping review
Source: Front Med (Lausanne). 2023 Apr 3;10:1148623. doi: 10.3389/fmed.2023.1148623 (PMC10106745; doi:10.3389/fmed.2023.1148623)
Supplement: Supplementary file 1 [file Data_Sheet_1.PDF]

Search strategies

1. **Scopus (23/01/2022)**

12 ((TITLE-ABS-KEY (((gene OR genes OR genetic) W/3 (therap* OR repair* OR correction*)))) OR (TITLE-ABS-KEY ("dna therap*"))) AND ((TITLE-ABS-KEY (osteoarthr*)) OR (TITLE-ABS-KEY (osteo-arthr*)) OR (TITLE-ABS-KEY (arthros*)) OR (TITLE-ABS-KEY ((degenerative W/3 arthriti*))) OR (TITLE-ABS-KEY ((degenerative W/3 joint* W/3 disease*))) OR (TITLE-ABS-KEY (coxarthros*)) OR (TITLE-ABS-KEY (gonarthros*))) 780 results

11 (TITLE-ABS-KEY (osteoarthr*)) OR (TITLE-ABS-KEY (osteo-arthr*)) OR (TITLE-ABS-KEY (arthros*)) OR (TITLE-ABS-KEY ((degenerative W/3 arthriti*))) OR (TITLE-ABS-KEY ((degenerative W/3 joint* W/3 disease*))) OR (TITLE-ABS-KEY (coxarthros*)) OR (TITLE-ABS-KEY (gonarthros*)) 207,522 results

10 TITLE-ABS-KEY (gonarthros*) 2,389 results

9 TITLE-ABS-KEY (coxarthros*) 2,451 results

8 TITLE-ABS-KEY ((degenerative W/3 joint* W/3 disease*)) 4,842 results

7 TITLE-ABS-KEY ((degenerative W/3 arthriti*)) 2,335 results

6 TITLE-ABS-KEY (arthros*) 69,366 results

5 TITLE-ABS-KEY (osteo-arthr*) 1,140 results

4 TITLE-ABS-KEY (osteoarthr*) 147,898 results

3 (TITLE-ABS-KEY (((gene OR genes OR genetic) W/3 (therap* OR repair* OR correction*)))) OR (TITLE-ABS-KEY ("dna therap*") 143,187 results

2 TITLE-ABS-KEY ("dna therap*") 116 results

1 TITLE-ABS-KEY (((gene OR genes OR genetic) W/3 (therap* OR repair* OR correction*))) 143,126 results

<u>Strategy in 1 equation</u>

((TITLE-ABS-KEY (((*gene* OR *genes* OR *genetic*) W/3 (*therap** OR *repair** OR *correction**)))) OR (TITLE-ABS-KEY (*"dna therap*"*))) AND ((TITLE-ABS-KEY (*osteoarthr**)) OR (TITLE-ABS-KEY (*osteo-arthr**)) OR (TITLE-ABS-KEY (*arthros**)) OR (TITLE-ABS-KEY ((*degenerative* W/3 *arthriti**))) OR (TITLE-ABS-KEY ((*degenerative* W/3 *joint** W/3 *disease**))) OR (TITLE-ABS-KEY (*coxarthros**)) OR (TITLE-ABS-KEY (*gonarthros**))) 780 results

Updated search strategy (March 2023): 66 document results

((TITLE-ABS-KEY (((gene OR genes OR genetic) W/3 (therap* OR repair* OR correction*)))) OR (TITLE-ABS-KEY ("dna therap*"))) AND ((TITLE-ABS-KEY (osteoarthr*)) OR (TITLE-ABS-KEY (osteo-arthr*)) OR (TITLE-ABS-KEY (arthros*)) OR (TITLE-ABS-KEY ((degenerative W/3 arthriti*))) OR (TITLE-ABS-KEY ((degenerative W/3 joint* W/3 disease*))) OR (TITLE-ABS-KEY (coxarthros*)) OR (TITLE-ABS-KEY (gonarthros*))) AND ORIG-LOAD-DATE AFT 20220123

2. Ovid Medline (March 02,2023)

Database: Ovid MEDLINE(R) ALL <1946 to March 02, 2023>

Search Strategy:

----

1 Genetic Therapy/ (52544)

2 Targeted gene repair/ (201)

3 ((gene or genes or genetic) adj3 (therap* or repair* or correction*)).ti,ab,kf. (82728)

4 DNA therap*.ti,ab,kf. (100)

5 1 or 2 or 3 or 4 (104404)

6 exp Osteoarthritis/ (75797)

7 osteoarthr*.ti,ab,kf. (92344)

8 osteo-arthr*.ti,ab,kf. (635)

9 arthros*.ti,ab,kf. (46096)

10 (degenerative adj3 arthriti*).ti,ab,kf. (1748)

11 (degenerative adj3 joint* adj3 disease*).ti,ab,kf. (3757)

12 coxarthros*.ti,ab,kf. (1705)

13 gonarthros*.ti,ab,kf. (1221)

14 6 or 7 or 8 or 9 or 10 or 11 or 12 or 13 (155956)

15 5 and 14 (450)

3. Embase (23/01/2022)

```
Embase
Session Results
........................................................
No.  Query Results                                          Results  Date
#26. #12 AND #25                                                800  23 Jan 2022
#25. #13 OR #14 OR #15 OR #16 OR #17 OR #18 OR #19 OR       211,642  23 Jan 2022
     #20 OR #21 OR #22 OR #23 OR #24
#24. gonarthros*:ti,ab,kw                                     1,771  23 Jan 2022
#23. coxarthros*:ti,ab,kw                                     2,218  23 Jan 2022
#22. (degenerative NEAR/3 joint* NEAR/3                       4,907  23 Jan 2022
     disease*):ti,ab,kw
#21. (degenerative NEAR/3 arthriti*):ti,ab,kw                 2,173  23 Jan 2022
#20. arthros*:ti,ab,kw                                       53,620  23 Jan 2022
#19. 'osteo arthr*':ti,ab,kw                                    828  23 Jan 2022
#18. osteoarthr*:ti,ab,kw                                   119,853  23 Jan 2022
#17. 'knee osteoarthritis'/de                                39,179  23 Jan 2022
#16. 'hip osteoarthritis'/de                                 13,267  23 Jan 2022
#15. 'hand osteoarthritis'/de                                 1,963  23 Jan 2022
#14. 'experimental osteoarthritis'/de                           367  23 Jan 2022
#13. 'osteoarthritis'/de                                     95,412  23 Jan 2022
#12. #1 OR #2 OR #3 OR #4 OR #5 OR #6 OR #7 OR #8 OR        138,918  23 Jan 2022
     #9 OR #10 OR #11
#11. 'dna therap*':ti,ab,kw                                     123  23 Jan 2022
#10. ((gene OR genes OR genetic) NEAR/3 (therap* OR         112,736  23 Jan 2022
     repair* OR correction*)):ti,ab,kw
#9.  'viral gene therapy'/de                                  7,910  23 Jan 2022
#8.  'stem cell gene therapy'/de                              1,215  23 Jan 2022
#7.  'somatic gene therapy'/de                                  293  23 Jan 2022
#6.  'nonviral gene therapy'/de                               2,675  23 Jan 2022
#5.  'gene replacement therapy'/de                            1,472  23 Jan 2022
#4.  'cell based gene therapy'/de                             1,621  23 Jan 2022
#3.  'antiangiogenic gene therapy'/de                           292  23 Jan 2022
#2.  'angiogenic gene therapy'/de                               249  23 Jan 2022
#1.  'gene therapy'/de                                       65,322  23 Jan 2022
........................................................
```

Updated search strategy (March 2023) : 86 document results

SEARCH QUERY

(('gene therapy'/de OR 'angiogenic gene therapy'/de OR 'antiangiogenic gene therapy'/de OR 'cell based gene therapy'/de OR 'gene replacement therapy'/de OR 'nonviral gene therapy'/de OR 'somatic gene therapy'/de OR 'stem cell gene therapy'/de OR 'viral gene therapy'/de OR ((gene OR genes OR genetic) NEAR/3 (therap* OR repair* OR correction*)):ti,ab,kw OR 'dna therap*':ti,ab,kw) AND ('osteoarthritis'/de OR 'experimental osteoarthritis'/de OR 'hand osteoarthritis'/de OR 'hip osteoarthritis'/de OR 'knee osteoarthritis'/de OR osteoarthr*:ti,ab,kw OR 'osteo arthr*':ti,ab,kw OR arthros*:ti,ab,kw OR

(degenerative NEAR/3 arthriti*):ti,ab,kw OR (degenerative NEAR/3 joint* NEAR/3 disease*):ti,ab,kw OR coxarthros*:ti,ab,kw OR gonarthros*:ti,ab,kw)) AND [23-01-2022]/sd NOT [04-03-2023]/sd
